# Supplementary figures and images for: A Bayesian phylogenetic hidden Markov model for B cell receptor sequence analysis
Source: PLoS Comput Biol. 2020 Aug 17;16(8):e1008030. doi: 10.1371/journal.pcbi.1008030 (PMC7451993; doi:10.1371/journal.pcbi.1008030)

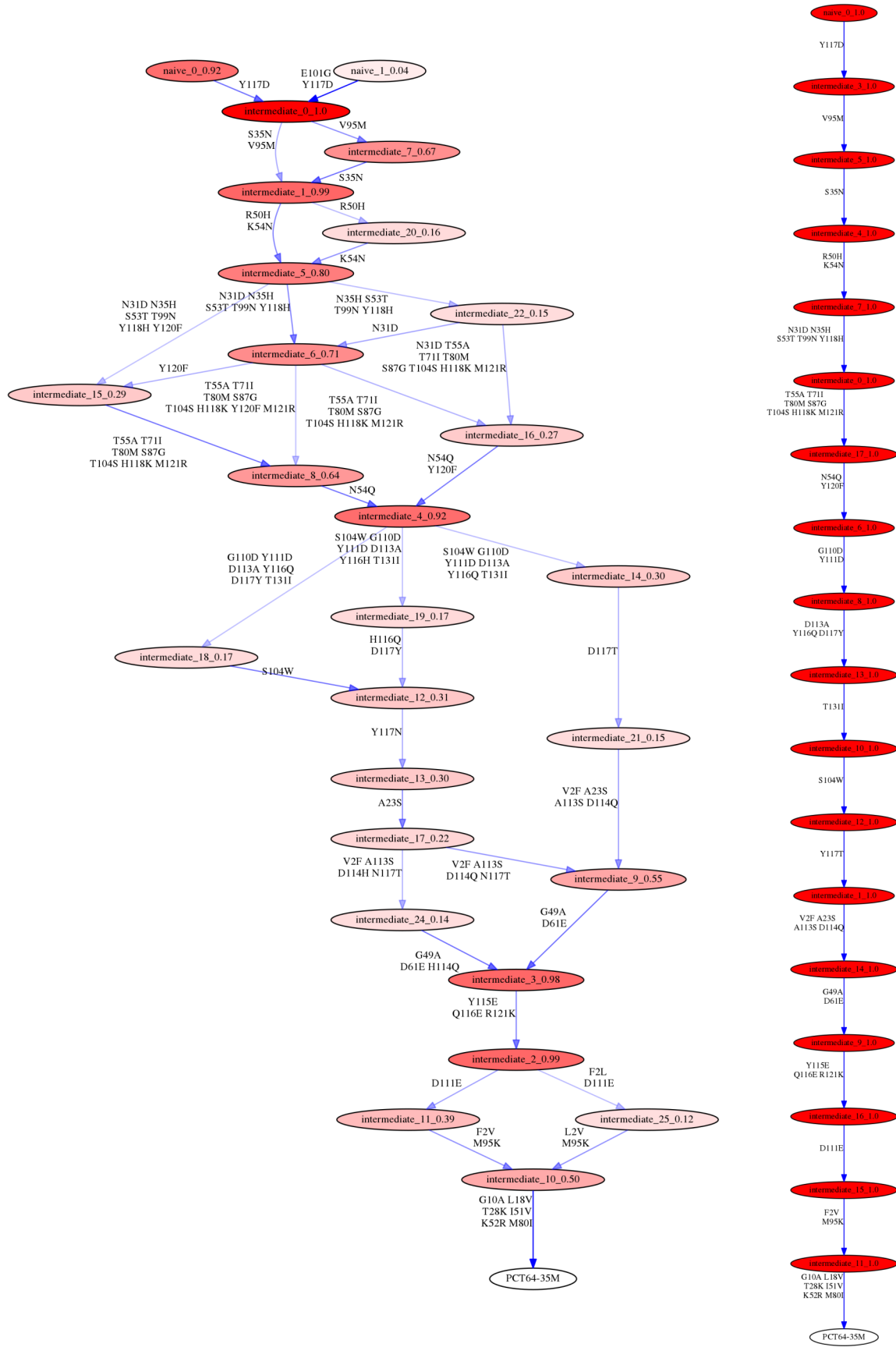

(a) PC64 posterior probability lineage inference

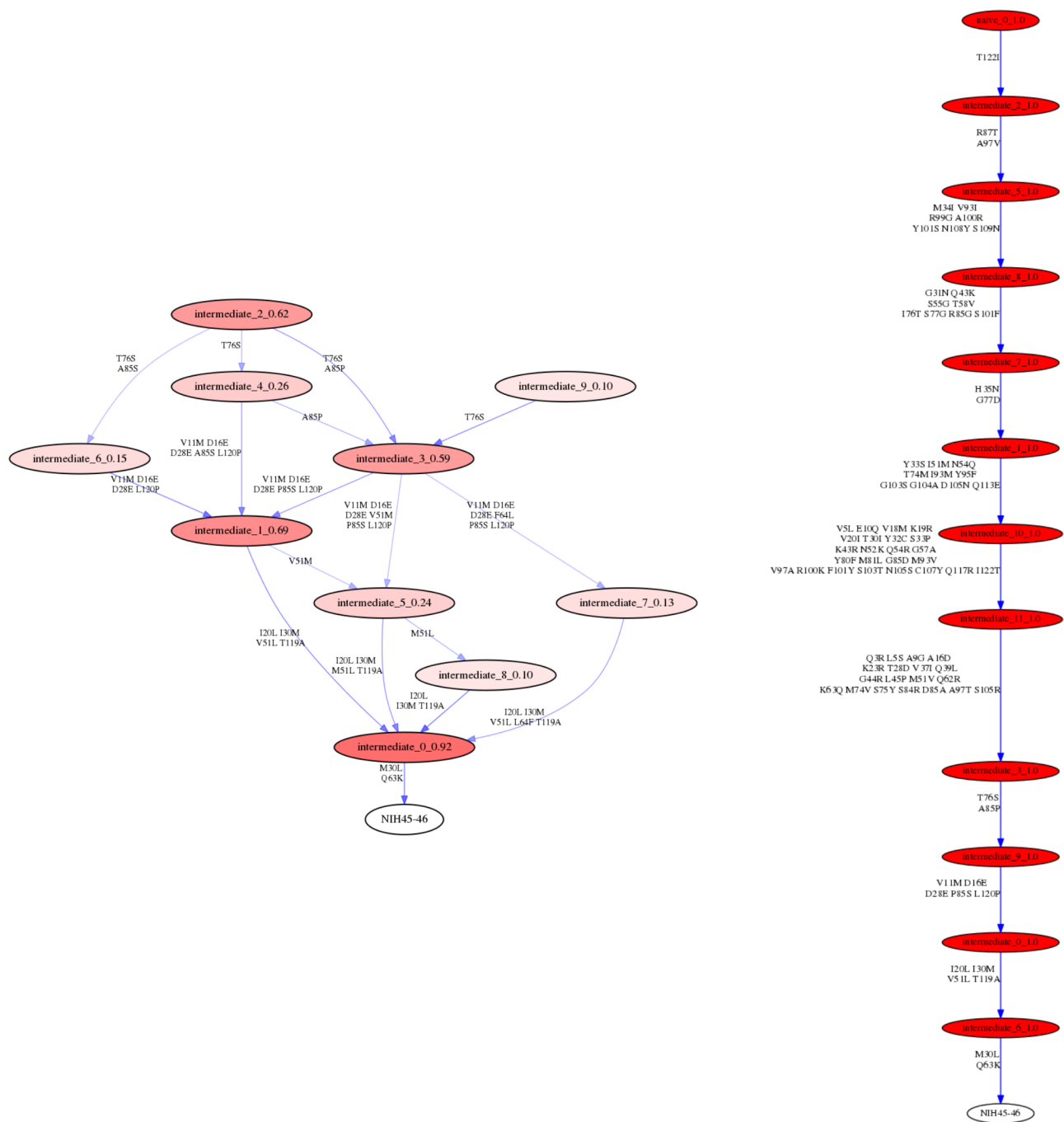

(b) VRC01 posterior probability lineage inference

Supplement: S1 Fig — The linearham-inferred (left) and dnaml-inferred (right) naive-to-tip amino acid sequence trajectories for the pruned PC64 dataset of 100 sequences and the trimmed VRC01 alignment of 268 sequences, displaying only the edges that satisfy the given posterior probability threshold, and only the nodes that contact edges above the threshold. The tip sequences of interest for the PC64 and VRC01 datasets are chosen to be PCT64-35M and NIH45-46, respectively, and we use 0.04 probability cutoffs for these lineage graphics (such that any edge with probability less than this threshold is discarded). The nodes correspond to unique ancestral sequences filled with red color, where the opacity is proportional to the posterior probability of the associated sequence. Each node has a label that denotes whether the associated sequence is a naive or intermediate ancestral sequence, the posterior probability rank of the sequence among all sampled naive or intermediate ancestral sequences, and the sequence-specific posterior probability itself. The directed edges connecting nodes represent ancestral sequence transitions, are shaded blue with an opacity proportional to the posterior probability of the associated sequence transition, and are annotated with the site-specific mutations between the two sequences. The absence of many nodes for VRC01 indicates that these naive-to-tip sequence trajectories are highly uncertain. (PDF) [file pcbi.1008030.s001.pdf]

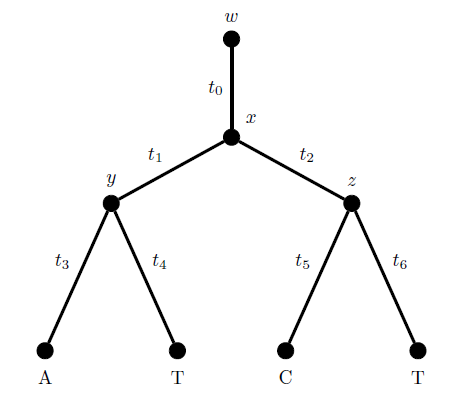

Supplement: S2 Fig — Letters x, y, z, w represent the unobserved internal node states where w is associated with the root node, t0 defines the root branch length, and (t0, t1, t2, …, t6) denotes the entire vector of branch lengths. Given this tree topology and set of branch lengths, we can calculate the likelihood of observing the nucleotide vector (A, T, C, T) by marginalizing probabilities over the unobserved states x, y, z, w. (PNG) [file pcbi.1008030.s002.PNG]

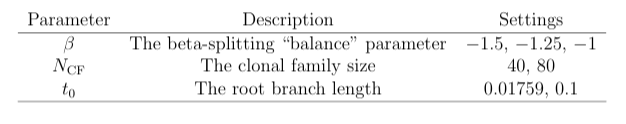

Supplement: S1 Table — A table summarizing the various parameter settings used in our simulation experiments. The beta-splitting “balance” parameter β controls how “comb-like” (i.e. imbalanced) our simulated trees look, NCF represents the number of sequences simulated in each clonal family, and t0 denotes the root branch length. We simulate 15 trees for each combination of parameter values, which results in 180 simulated trees. (PNG) [file pcbi.1008030.s003.PNG]

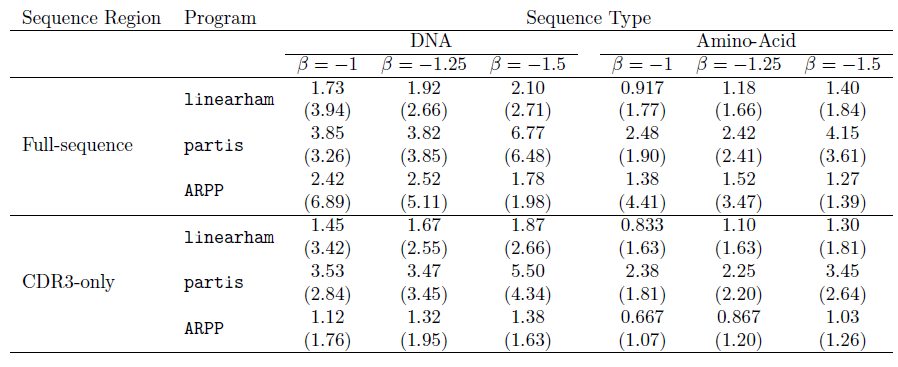

Supplement: S2 Table — Mean hamming distances between the simulated naive sequences and their corresponding estimates, where the hamming distances are averaged over all trees generated under the different beta-splitting “balance” parameter value settings. Results are provided for the linearham, partis, and ARPP programs; the full-sequence and CDR3 regions; and the DNA/amino-acid sequence types. Standard errors are also presented in parentheses. (PNG) [file pcbi.1008030.s004.PNG]

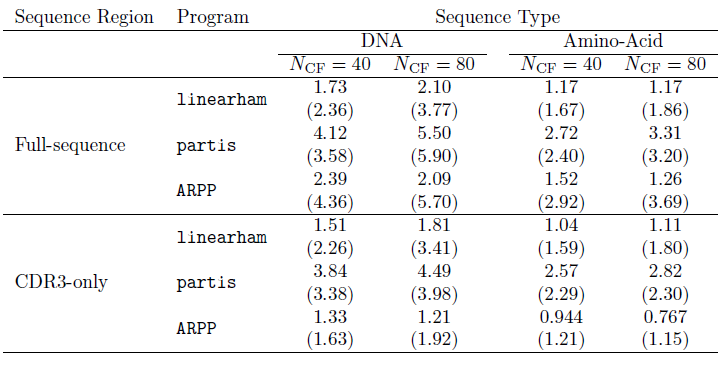

Supplement: S3 Table — Table analogous to S2 Table, but varying the CF sequence count NCF. (PNG) [file pcbi.1008030.s005.PNG]

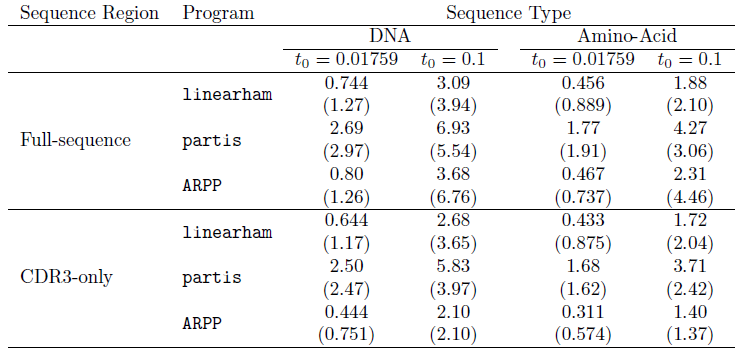

Supplement: S4 Table — Table analogous to S2 Table, but varying the root branch length t0. (PNG) [file pcbi.1008030.s006.PNG]

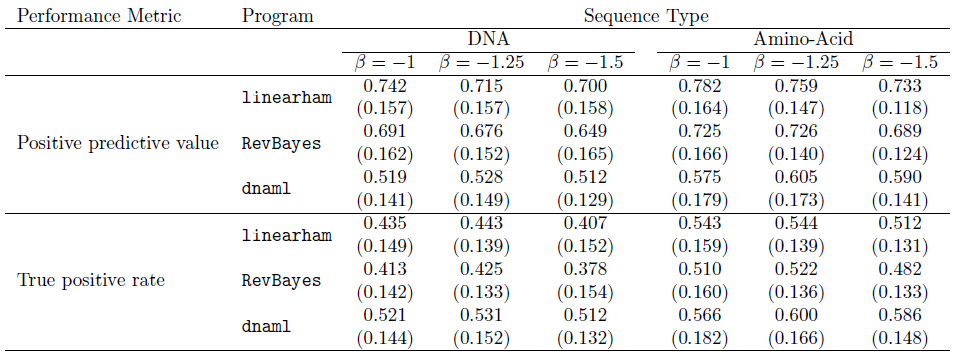

Supplement: S5 Table — Mean positive predictive values and mean true positive rates for decision boundary ρ = 0.5, where we average over all trees generated under the different beta-splitting “balance” parameter value settings. Results are provided for the linearham, RevBayes, and dnaml programs and the DNA/amino-acid sequence types. Standard errors are also presented in parentheses. (PNG) [file pcbi.1008030.s007.PNG]

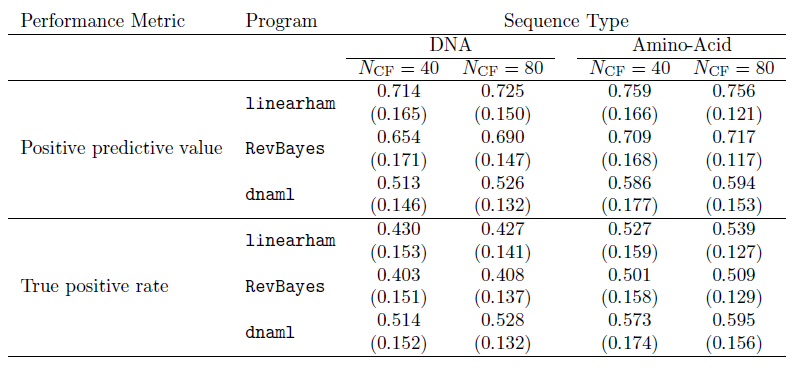

Supplement: S6 Table — Table analogous to S5 Table, but varying the CF sequence count NCF. (PNG) [file pcbi.1008030.s008.PNG]

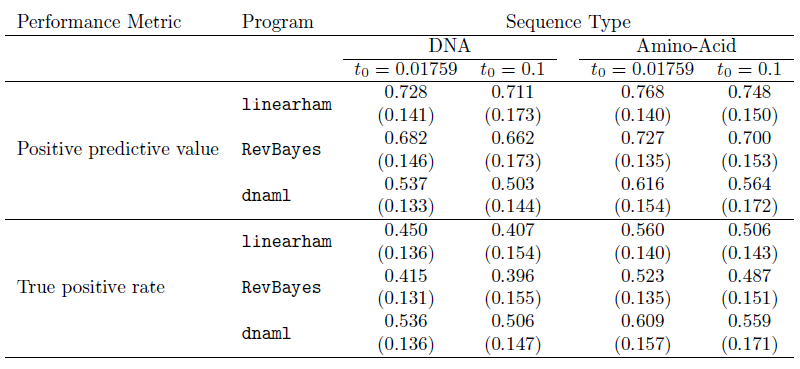

Supplement: S7 Table — Table analogous to S5 Table, but varying the root branch length t0. (PNG) [file pcbi.1008030.s009.PNG]
